# Dilute Mitrate Acid in Constitutional Syphilis

**Published:** 1854-08

**Authors:** 


*Dilute Nitric Acid in Constitutional Syphilis.*—A practical rule of great value in the management of the constitutional forms of syphilitic disease is never to persevere with a remedy which, after a fair trial, produces no apparent benefit. It is a disease which, if the right treatment be hit upon, generally yields with surprising rapidity. Another rule which we observe most practical surgeons adopt, is, to change the remedy, if, after great improvement, the affection come to a stand-still or appear to retrograde. By ringing changes on the iodide of potassium, small doses of the bichloride, iodide, or biniodide of mercury, or the dilute nitric acid, much greater good may be done, than by the continuous employment of any one of these preparations. The dilute nitric acid is a drug which has a considerable reputation in this disease, but of which the exact effects have not been sufficiently investigated. Whether it acts merely as a tonic or alterative, or whether it has specific power over the disease, is yet a debated question. The following case, in which, after the iodide had failed, it produced effects which might seem to denote a specific influence, is worthy of narration. We epitomize it from the daily record kept by Mr. Edmonds, the dresser of the patients:

E. J., aged 20, a prostitute, was admitted into St. Thomas's, under Mr. Simon's care, on April 26, suffering severely from an eruption of ulcerating rupia, the large sores formed by which were scattered over the thighs, nates, and some other parts of the body. The disease had marked syphilitic characters, and she admitted having been salivated for the original affection one year previously. Mr. Simon ordered a meat diet with eight ounces of wine daily; the sores to be dressed with a bichloride lotion (gr. ij. ad ℥j.), and a draught containing four grains of iodide of potassium dissolved in water, to be taken three times daily. These measures were continued for ten days without benefit, the patient meanwhile taking her food well. On May 6, some of the sores were decidedly increasing, and one of the larger was in a very sloughy state. The draught was now changed for

℞ Acid. nitric dilute ♏xxv.,

Syrupi ʒij.,

Quin. disulph. gr. ii.,

Aquæ ℥j.

The same lotion was continued, the quantity of wine increased to twelve ounces, and two eggs added to the daily allowance of food. A surprisingly rapid improvement ensued on this change of measures. The sores at once cleaned and commenced to heal. In the course of ten days several were almost cicatrized, and the patient is now so nearly well that she will shortly be discharged. It is possible, of course, that the quinine in this case, which was commenced simultaneously with the acid,.had some share in the cure. As, however, a tonic regimen, with wine, had been pursued before, without the least benefit, it seems fair to attribute the chief influence to the latter.—*London Medical Times.*

